# Assessing the impact of malignant thyroid nodules on the efficacy of radiofrequency ablation: a single-center prospective study

**DOI:** 10.3389/fendo.2025.1669194

**Published:** 2025-10-13

**Authors:** Huan Liu, Chuanke Shi, Zhideng Yan, Ming Luo

**Affiliations:** Department of General Surgery, Zhongshan Hospital of Traditional Chinese Medicine, Zhongshan, Guangdong, China

**Keywords:** radiofrequency ablation (RFA), efficacy, thyroid nodules (TNs), papillary thyroid carcinoma, risk factors

## Abstract

**Background:**

Radiofrequency ablation (RFA) is currently the mainstay of treatment for thyroid nodules (TNs), with indications that extend from initially benign nodules and micropapillary thyroid carcinoma to current low-risk papillary thyroid carcinoma. Our study aims to evaluate the impact of malignant nodules on the efficacy of RFA.

**Methods:**

A total of 970 eligible patients were divided into benign and malignant group. We analyzed whether the efficacy of RFA differed between the two groups in terms of TNs volume, volume reduction ratio (VRR), technical effectiveness (TE), complete ablation (CR), and regrowth rate, and used multivariate logistic and linear regression to assess whether malignant nodules were a risk factor for its efficacy.

**Results:**

The TE value was higher in the benign group than in the malignant group (81.7% vs. 70.5%, p=0.002 < 0.052). However, after excluding patients with less than 1 year of follow-up, the adjusted TE values were not significantly different between the two groups. The malignant group had a higher CR than the benign group (43.6% vs. 9.7%, p < 0.001) and its CR time was shorter (14.09 ± 11.50 months vs. 21.75 ± 13.18 months, p < 0.001). The regrowth rate was higher in the benign group than in the malignant group. Multivariate logistic analysis showed that there was no difference between the two groups in TE and regrowth, however, the benign group had a lower CR than the malignant (adjusted OR = 0.100, 95%CI 0.055- 0.181, P<0.001). Multivariate linear regression analysis showed that the VRR in the malignant group was lower than that in the benign at years 1 and 2 after RFA, but there was no difference after 2 years.

**Conclusion:**

Malignant thyroid nodules do not affect the efficacy of radiofrequency ablation. Although their early target regression is slower after RFA, there is no difference in long-term outcomes, and they are more likely to achieve complete regression.

## 1 Introduction

Thyroid nodules (TNs) are extremely common diseases in the general population and their incidence is increasing. Surgery is currently the mainstay of treatment for TNs (1), and radiofrequency ablation (RFA) is an alternative approach. Many studies have confirmed that the efficacy of RFA for TNs is not significantly different from that of surgery; however, its risk of intraoperative and postoperative adverse events is significantly lower than that of surgery (2). RFA was first reported for the treatment of benign thyroid nodules in 2006 (3), and its use now extends to large benign nodules, micropapillary thyroid cancer, and even recurrent and low-risk thyroid cancer. As its indications have expanded, factors affecting its efficacy have increased. Volume reduction ratio (VRR) and regrowth after RFA are two important metrics to assess their efficacy. Sim et al. found that initial nodule volume, vascularity, and technical factors were associated with its efficacy (4). Yan L et al. also demonstrated that initial volume, vascularity, and nodule location affect the efficacy of RFA (5). One study found that the initial ablation ratio (IAR) was significantly associated with the likelihood of VRR, but not with nodule regrowth (6). Bernardi et al. found that the energy delivered during RFA is significantly correlated with regrowth (7), while Aljammal Jules et al. found that it is negatively correlated with VRR (8).

The popularity of the RFA application and the expansion of its indications have resulted in many new clinical concerns. Previous studies have showed that RFA does not affect subsequent re-RFA or surgical procedures for TNs (9); however, whether it affects the efficacy of subsequent RFA for residual nodules remains unclear. Due to the trend towards younger patients and the increasing importance of aesthetic concerns, more and more patients with micropapillary thyroid cancer, or even low-risk thyroid cancer, are opting for RFA treatment. Previous studies have found that the VRR of malignant nodules after RFA is significantly lower than that of benign nodules (10), but there are currently few studies on the effect of malignant nodules on the efficacy of RFA.

Therefore, the aim of our study was to explore whether malignant nodules affect the efficacy of RFA.

## 2 Materials and methods

### 2.1 Patients and groups

Our study was a single-center prospective cohort study, which was approved by our Institutional Ethics Committee (No. 2022ZSY-LLK-456), and all patients obtained informed consent prior to RFA. We enrolled patients with TNs who underwent RFA between January 2017 and December 2022 at our institution. All thyroid nodules were assessed for malignancy risk before RFA by experienced sonographers according to the ACR TI-RADS grading system (11). All patients underwent fine needle aspiration biopsy (FNA) before RFA to clarify their pathology.

The inclusion criteria for eligible patients were as follows: 1) RFA performed at our institution between January 2017 and December 2022; 2) TNs with TI-RADS grading; and 3) TNs with definitive FNA pathology results. The exclusion criteria were as follows: 1) those who refused to participate; 2) patients who were lost to follow-up; 3) incomplete data; and 4) those who received other thermal ablation treatments before RFA, including laser ablation (LA), microwave ablation (MA), and high-frequency focused ultrasound (HIFU). All the above eligible patients were divided into benign group and malignant group based on TNs pathology. The malignant group were all papillary thyroid carcinomas and there was no evidence of capsule rupture or invasion of surrounding tissues on neck ultrasound examination. In addition, there is no evidence of cervical lymph node enlargement on neck ultrasound or Computed Tomography (CT) scan.

### 2.2 RFA procedure

All RFAs were performed by experienced surgeons and sonographers in an outpatient setting. The patient was placed in the supine position with full neck extension, and local anesthesia with lidocaine was applied for pain control. The RFA was performed under real-time ultrasound guidance with hydrodissection, trans-isthmic approach, and the moving shot technique. Hydrodissection involves injecting a 5% glucose solution in the perithyroidal capsule prior to RFA to form a liquid isolation zone greater than 5 mm, thereby avoiding damage to the recurrent laryngeal nerve, superior laryngeal nerve, trachea, esophagus, and other structures. During the injection of the liquid isolation zone, 5–20 ml of liquid is injected at a time, and multiple injections can be administered as needed during the procedure. All RFAs were performed using bipolar radiofrequency ablation needles with an 18G diameter, 10 cm length, and 0.9 or 2 cm active tip, with an energy delivered of 4–8 W, based on the size and location of the thyroid nodule. To ensure complete ablation of TNs, we determined the ablation area using contrast-enhanced ultrasound (CEUS) prior to the procedure, performed radiofrequency ablation under real-time ultrasound guidance, and repeated CEUS after the procedure to confirm complete ablation. During the RFA procedure, we assessed the patients for changes in voice, dyspnea, and other discomforts, and discharged them after 12 hours of post-operation observation without significant discomfort.

### 2.3 Post-RFA follow-up and variables

After RFA, ultrasound (US) was performed at months 1, 3, 6, 12, and every 6 or 12 months thereafter. Thyroid function was assessed again 1 month after RFA. We followed up to assess whether serious adverse events such as permanent nerve damage (including the superior laryngeal nerve, the recurrent laryngeal nerve, and the vagus nerve), or events requiring emergency surgery or prolonged hospitalization occurred after RFA.

We collected data on general demographic characteristics, and clinical characteristics of TNs preoperatively and postoperatively. General demographic characteristics included age, sex, and history of thyroid surgery and radiofrequency ablation. Preoperative clinical characteristics of TNs included their volume and TI-RADS classification. The clinical characteristics evaluated after RFA consisted of volume reduction ratio (VRR), technical efficiency (TE), regrowth rate, and reintervention rate. The three-dimensional size of the TNs was measured in ultrasound and its volume was calculated: Volume equation=[length(sagittal, cm)×depth(anteroposterior, cm)×width(transverse, cm)]×0.524. VRR = [(Initial Volume - Final Volume)×100%]/Initial Volume. We understand that in treatment of operable cancer the goal is to completely remove tumor in its entirety but to facilitate comparison with benign thyroid nodule we choose the technical efficacy as defined by a >50% reduction in thyroid nodule volume within 12 months after to be a main parameter. TNs regrowth was defined as a 50% increase in total volume over the previous minimum volume (12).

### 2.4 Statistical analysis

In our study, continuous variables were described by mean ± standard deviation (SD) and statistically analyzed by Students to test or Mann Whitney U test according to their distribution. Categorical variables are expressed in frequency (percentage), and statistical analysis is performed using Chi-square tests or Fisher exact tests when appropriate. We used multivariate logistic and linear regression analysis to find the factors that affected the RFA effect, expressed by the adjusted odds ratio (OR) and 95% confidence interval (CI). P < 0.05 was considered statistically significant. All data were analyzed using SPSS 25.0 version.

## 3 Results

**Figure 1:**
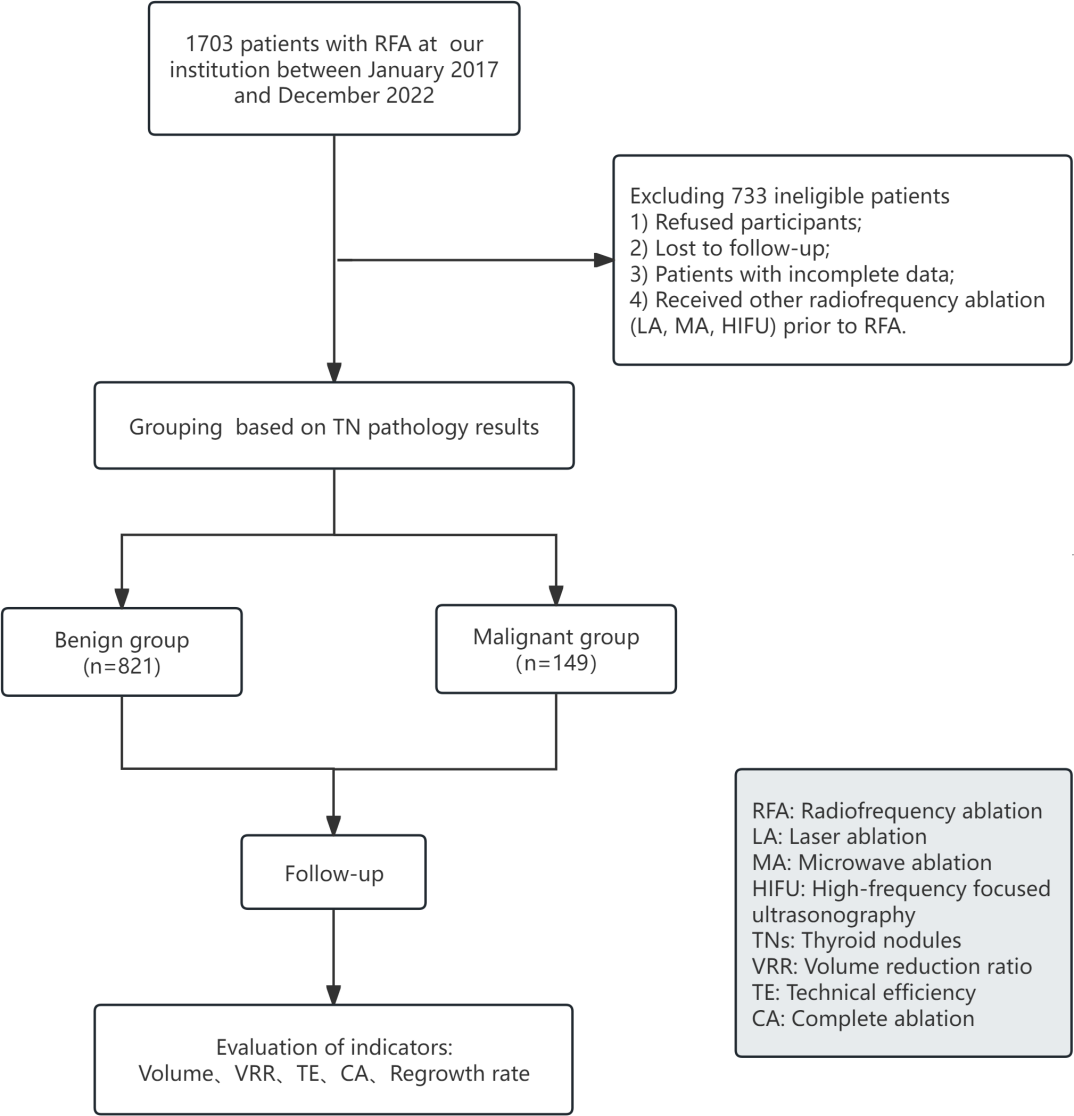
Flowchart of the study.

The flow of our study is shown in **Figure 1**. A total of 1,703 patients underwent RFA from January 2017 to December 2022, excluding refusers, loss to follow-up, incomplete data, and patients who underwent other ablations (LA, MA, HIFU) prior to RFA, for a total of 970 eligible patients divided into the benign group (n=821) and malignant group (n=149). The demographic and clinical characteristics of all patients were shown in **Table 1**. There were 803 (82.8%) females and 167 (17.2%) males with a mean age of 43.63 ± 12.36 years. Among these patients, 201 (20.7%) patients ablated multiple nodules during the same RFA, 18 (1.9%) patients underwent multiple RFAs and 15 (1.5%) patients were postoperative residual thyroid.

**Table 1:** Clinical characteristics of the study.

Characteristic	n (%)
Genders	
Female/male n (%)	803/167 (82.8%/17.2%)
Age, years	
(mean ± SD)	(43.63±12.36)
Number of TNs	
1/>1 n (%)	(316/654) (32.6%/67.4%)
The number of TNs for RFA	
1/>1 n (%)	(769/201) (79.3%/20.7%)
History of prior RFA	
Yes/no n (%)	(18/952) (1.9%/98.1%)
Postoperative residual thyroid	
Yes/no n (%)	(15/955) (1.5%/98.5%)
Pathologic results of TNs	
Benign/Malignant n (%)	(821/149) (84.6%/15.4%)

SD, Standard deviation; TNs, Thyroid nodules; RFA, radiofrequency ablation.

The demographic and clinical characteristics of the benign and malignant groups are shown in **Table 2**. The follow-up time in the benign group and malignant group was 18.10 ± 13.80 months and 15.30 ± 12.61 months, respectively. There were significantly more males in the malignant group than in the benign group (24.2% vs 16.0%, p=0.015<0.05). There were no significant differences between the two groups in the number of TNs, the number of TNs with RFA, the proportion of multiple RFAs, and the proportion of postoperative residual thyroid.

**Table 2:** Demographic and clinical characteristics of the two groups.

Variable	Benign group	Malignant group	P value
N (%)	821 (84.6)	14 9(15.4)	–
Follow-up time, months			
(mean ± SD)	18.10 ± 13.80	15.30 ± 12.61	0.021
Genders			
Female n (%)	690 (84.0)	11 3(75.8)	0.015
Male n (%)	131(16.0)	36 (24.2)	
Age, years			
(mean ± SD)	43.82 ± 12.44	42.56 ± 11.90	0.252
Number of TNs			
1 n (%)	258 (31.4)	58 (38.9)	0.072
>1 n (%)	563 (68.6)	91 (61.1)	
The number of TNs for RFA			
1 n (%)	650 (79.2)	11 9(79.9)	0.848
>1 n (%)	171 (20.8)	30(20.1)	
History of prior RFA			
Yes n (%)	804 (97.9)	148 (99.3)	0.244
no n (%)	17(2.1)	1 (0.7)	
Postoperative residual thyroid			
Yes n (%)	809 (98.5)	146 (98.0)	0.616
no n (%)	12 (1.5)	3 (2.0)	

SD, Standard deviation; TNs, Thyroid nodules; RFA, radiofrequency ablation.

The efficacy indicators after RFA were shown in **Tables 3**, **4**. The technical efficiency (TE) was higher in the benign group than in the malignant group (81.7% vs. 70.5%, p=0.002 < 0.05). TE was defined as a volume reduction of >50% at 1 year after RFA, the validity of which would be confounded by less than 1 year of follow-up. Excluding those patients who had less than 1 year of follow-up and did not achieve a volume reduction of at least 50%, we saw no statistically significant difference in adjusted TE between the two groups (89.5% vs. 84.7%, p=0.117 > 0.05). Complete ablation (CA) rates were significantly higher in the malignant group than in the benign group (43.6% vs. 9.7%, p < 0.001). The time to CA was longer in the benign group than in the malignant group (21.75 ± 13.18 months vs. 14.09 ± 11.50 months, p < 0.001). Regrowth occurred in 15 patients in the benign group and none in the malignant group, and its regrowth rate did not differ between the two groups (1.8% vs 0%, p=0.096 > 0.05). The initial volume of TNs was significantly greater in the benign group than in the malignant group (7.33 ± 8.36 ml vs. 0.19 ± 0.49 ml; p < 0.001). VRR at year 1 after RFA was significantly greater in the benign group than in the malignant group (58.78 ± 49.47% vs -8.41% ± 251.29%, p=0.003 < 0.05), however, it was not statistically different between the two groups after 1 year.

**Table 3:** The efficacy of RFA in the two groups.

Variable	Benign group	Malignant group	P value
TE			
Yes n (%)	671 (81.7)	105 (70.5)	0.002
No n (%)	150 (18.3)	44(29.5)	
TE*			
Yes n (%)	671 (89.5)	105 (84.7)	0.117
No n (%)	79 (10.5)	19 (15.3)	
CA rate			
Yes n (%)	80 (9.7)	65 (43.6)	<0.001
No n (%)	741 (90.3)	84 (56.4)	
CA time, months			
(mean ± SD)	21.75±13.18	14.09±11.50	<0.001
Regrowth rate			
Yes n (%)	15 (1.8)	0 (0.0)	0.096
No n (%)	806(98.2)	149 (100.0)	
Regrowth time,months			
(mean ± SD)	29.80±12.47	–	–

TE, technical efficiency; TE*, The adjusted technical effectiveness; CA, Complete ablation; SD, Standard deviation.

**Table 4:** Post RFA volume and VRR in both groups.

Variable	Volumes (ml)		P value	VRR (%)		P value
	Benign group	Malignant group		Benign group	Malignant group	
Before RFA procedure	7.33 ± 8.36	0.19 ± 0.49	<0.001	–	–	–
After RFA procedure						
12months	2.11 ± 2.81	0.14 ± 0.34	<0.001	58.78 ± 49.47	-8.41 ± 251.29	0.003
24months	1.86 ± 7.10	0.09 ± 0.22	0.091	74.03 ± 55.96	61.46 ± 47.32	0.106
36months	2.10 ± 8.61	0.06 ± 0.10	0.250	76.54 ± 30.88	70.30 ± 41.45	0.388
48months	1.67 ± 2.44	0.01 ± 0.01	<0.001	75.30 ± 32.31	59.60 ± 66.64	0.591
>48months	0.98 ± 1.49	0.01 ± 0.02	<0.001	84.21 ± 19.62	94.01 ± 7.48	0.334

RFA, Radiofrequency ablation; VRR, Volume reduction ratio; ml, Milliliter.

**Figure 2:**
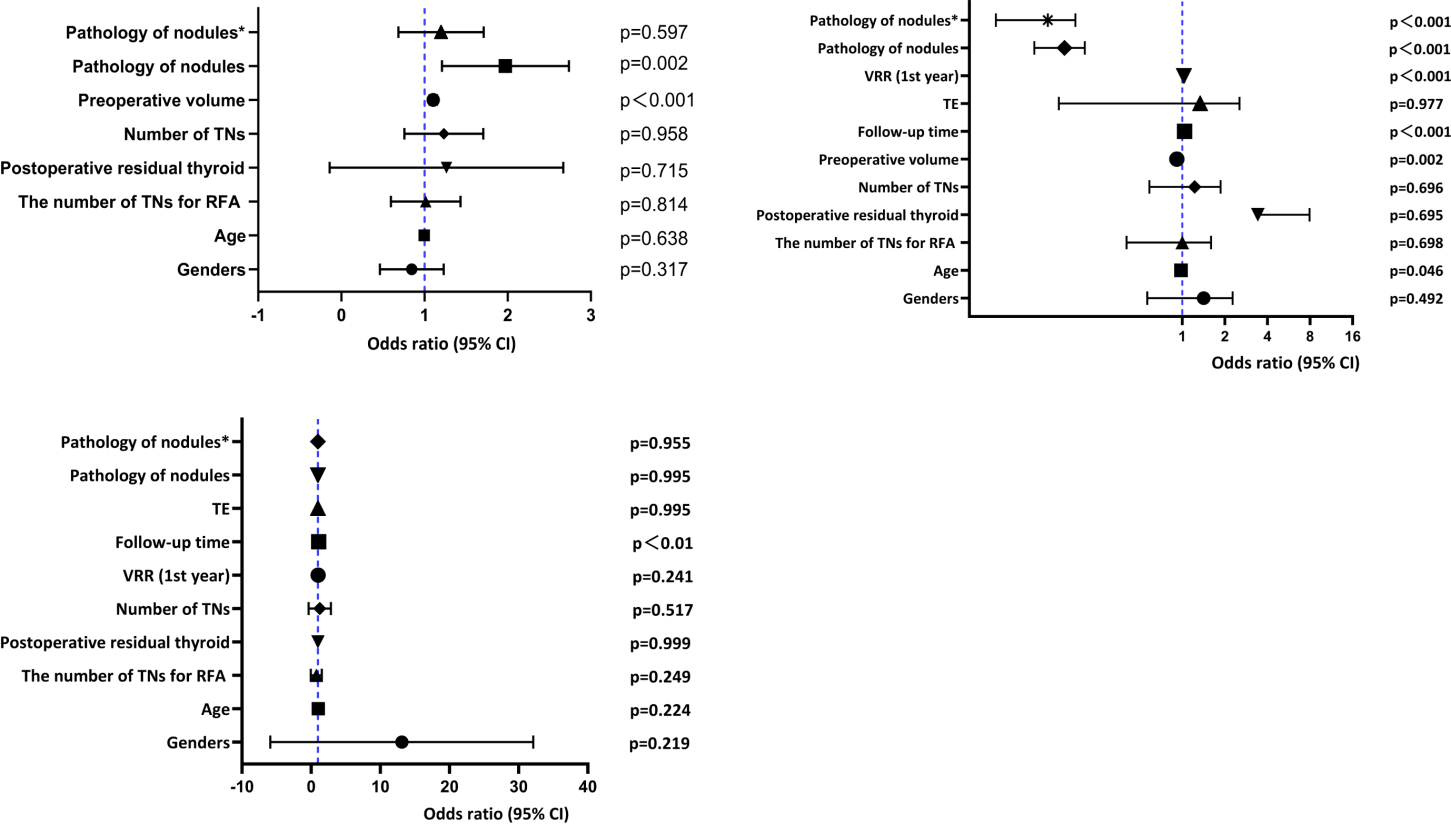
Multivariate logistic regression for TE.

**Figure 3:**
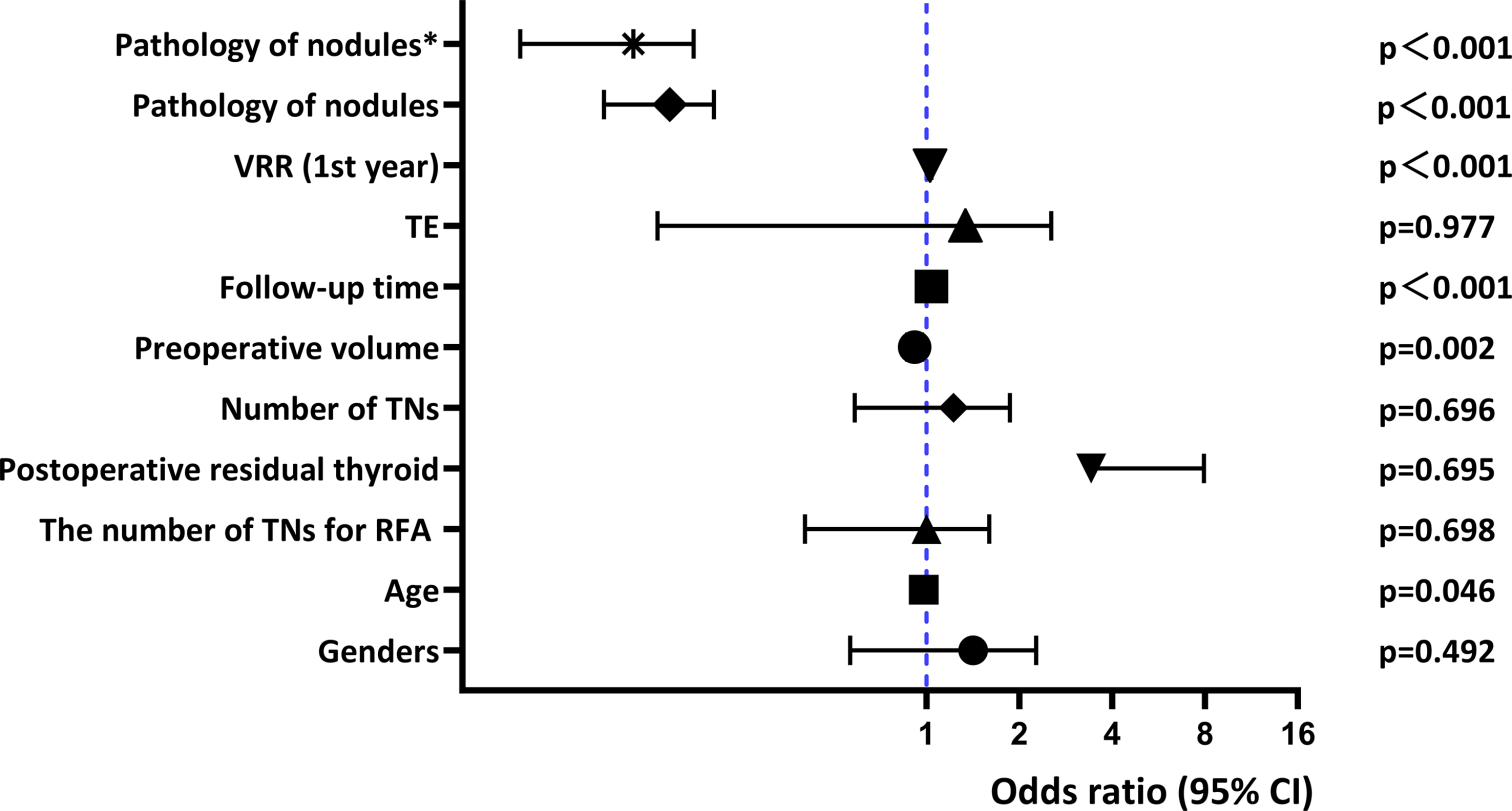
Multivariate logistic regression for CR.

**Figure 4:**
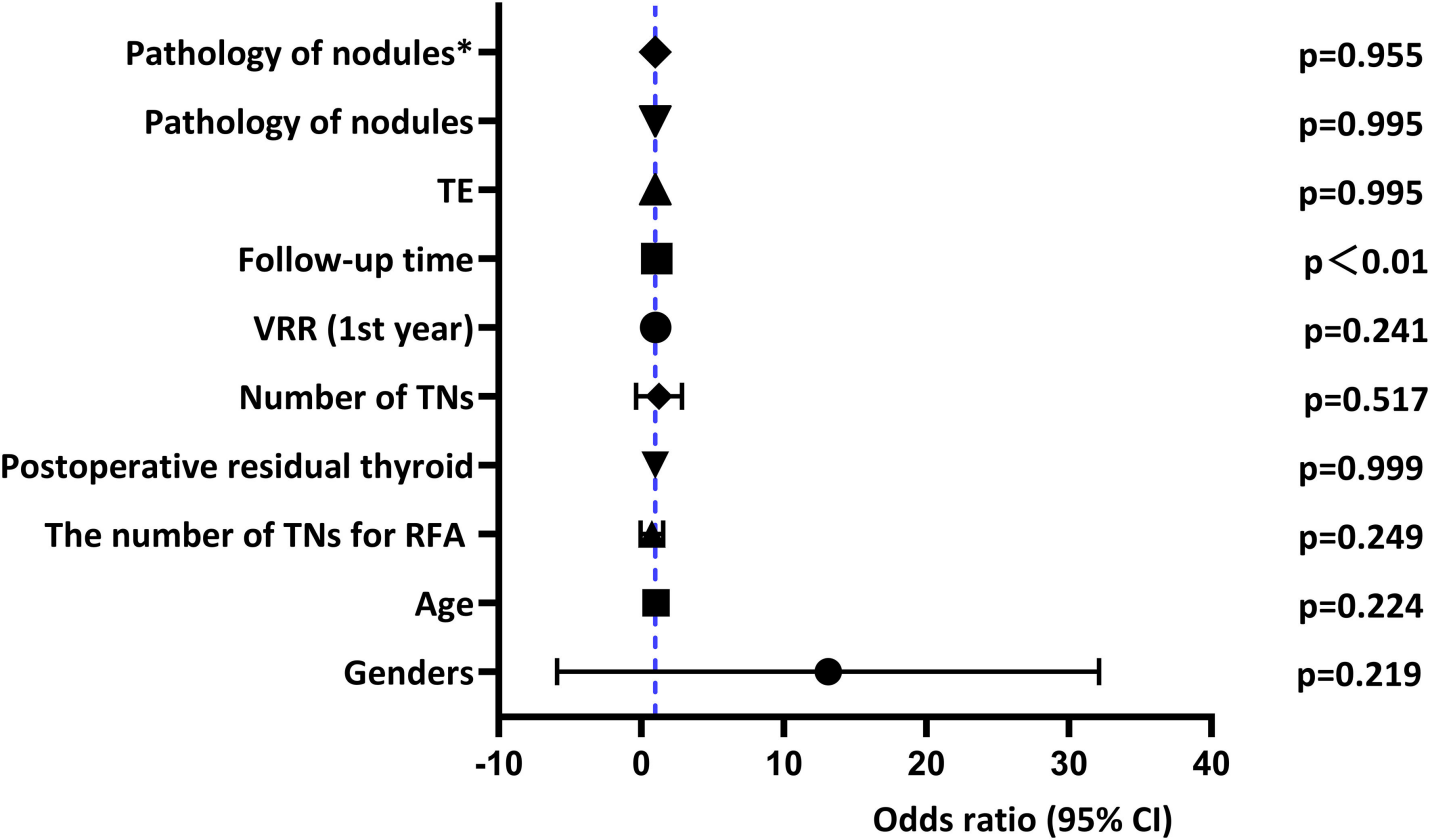
Multivariate logistic regression for regrowth.

The effect of malignant nodules on RFA efficacy is shown in **Figures 2**, **3** and **4** and **Table 5**. Risk factors for TE, CA, and regrowth after RFA are shown in **Figures 2**, **3** and **4**. In terms of TE, it was associated with malignant nodules (OR = 1.875, 95%CI 1.264-2.780,P=0.002<0.05) and preoperative volume (OR = 1.105, 95%CI 1.061-1.150,P<0.001). However, we found no statistical correlation between malignant nodules and TE using multivariate logistic regression analysis to exclude confounders (adjusted OR = 1.125, 95%CI 0.726-1.743, P = 0.597>0.05). In terms of CR, it was significantly associated with malignant nodules (OR = 0.140, 95%CI 0.094-0.208, P<0.001), follow-up time (OR = 1.037, 95%CI 1.019-1.056,P<0.001), preoperative volume (OR = 0.916, 95%CI 0.867-0.968,P=0.002< 0.05), and 1-year VRR (OR = 1.029, 95%CI 1.018-1.040,P<0.001). We used multivariate logistic regression analysis excluding these factors above and found that malignant nodules remained statistically associated with CR (adjusted OR = 0.100, 95%CI 0.055-0.181, P<0.001). In terms of growth, it did not correlate with malignant nodules (OR = 1, P = 0.995 > 0.05).In our study, we used multiple linear regression analysis to find that malignant nodules affected volume after RFA at years 1 and 2 (p ≤ 0.001; p ≤ 0.001), but not after 2 years. Moreover, it was similar for VRR (p≤ 0.001; p=0.037<0.05). Our analysis indicated that the trend of malignant nodule volume reduction was less than that of benign nodules within two years after RFA, and there was no significant difference after two years.

**Table 5:** Impact of malignant nodules on volume and VRR after RFA.

Postoperative follow-up (p-value)						
Variable	1 year	2 years	3 years	4 years	5 years	>5 years
Volumes	≤0.001	≤0.001	0.184	0.569	0.692	0.368
VRR	≤0.001	0.037	0.199	0.819	0.397	0.098

VRR, Volume reduction ratio; RFA, Radiofrequency ablation.

## 4 Discussion

Radiofrequency ablation is currently an alternative treatment for thyroid nodules, and many studies have confirmed its favorable efficacy and safety (13–15). The indications for RFA are benign nodules ranging from initially small to giant nodules, and malignant nodules ranging from initially micropapillary thyroid cancer to low-risk thyroid cancer. Thyroid nodules undergo coagulative necrosis after RFA, which appears as high echogenicity similar to calcification on ultrasound examination. However, malignant nodules typically appear on ultrasound as poorly defined with peripheral calcification, which may affect the determination of the radiofrequency ablation range.

Koike E et al. found the incidence of calcification to be 8%-32% and 26%-54% in benign and malignant nodules, respectively (16). Park KW et al. also indicated that large calcifications may lead to RFA treatment failure (17). Most of the calcifications are concentrated in the periphery, which can diminish the thermal effect of RFA; these may limit complete ablation of the peripheral. Wu MH et al. showed that TNs after RFA were more likely to be characterized by calcification, heterogeneity, and ill-defined margin (18). Many studies have found that the majority of recurrences after RFA are peripheral and have also confirmed that incomplete ablation of the periphery is an important risk factor for recurrence (5, 19, 20). Sim JS et al. found that re-expansion of the margins after RFA was an important cause of recurrence (22). In our study, we saw that malignant nodules affected the technical efficiency of RFA (70.5% vs. 81.7%, p=0.002 < 0.05); however, after excluding other confounders, it was found that malignant nodules did not (84.7% vs. 89.5%, p=0.117 > 0.05). The CA rate was significantly more favorable in the malignant group than in the benign group (43.6% vs. 9.7%, p < 0.001 < 0.05), and the mean time to CA was significantly shorter in the malignant group than in the benign group (14.09 ± 11.50 months vs. 21.75 ± 13.18 months, p < 0.001 < 0.05). Our study showed that benign nodules were less likely to achieve CR than malignant nodules (OR = 0.140, 95%CI 0.094-0.208, P<0.001). These differences may be due to the proportions of their calcifications, as benign nodules usually show patchy, eggshell-like calcifications, whereas malignant nodules usually have diffuse loculated calcifications. These differences could also be due to the size of the nodules, as the volume of the benign group was significantly larger than that of the malignant group (7.33 ± 8.36 ml vs 0.19 ± 0.49 ml, p < 0.001). The regrowth rate was higher in the benign group than in the malignant group, but it was not statistically significant (1.8% vs 0%, p=0.096 > 0.05). Yan L et al. found that initial volume was an independent risk factor for regrowth of benign nodules after RFA (OR = 1.047, 95%CI 1.020-1.075) (21), which is consistent with several studies (23, 24). One study also found that VRR at 12 months after RFA was associated with regrowth (25).

Volume and VRR after RFA are important indicators to assess its efficacy. In our study, the initial volume was significantly greater in the benign group than in the malignant group (7.33 ± 8.36 ml vs 0.19 ± 0.49 ml, p < 0.001 < 0.05). VRR at year 1 after RFA was significantly greater in the benign group than in the malignant group (58.78 ± 49.47% vs -8.41% ± 251.29%, p=0.003 < 0.05), however, it was not statistically different between the two groups after 1 year. We have seen an increase in the volume of malignant nodules rather than a decrease in volume in the 1st year after RFA, which may be caused by the fact that during RFA to ensure complete ablation of the nodule, we usually ablate beyond the nodule border and, moreover, the initial volume of the malignant nodule is smaller. Lin WC et al. found that VRR at 1 month after RFA was better in large nodules (volume >30 ml) than in small nodules (volume <10 ml) (47.13 ± 21.51% vs. 30.25 ± 70.10%, p <0.001 < 0.05), which is consistent with our study (26). We saw that malignant nodules were smaller than benign nodules after year 4, but there was a significant difference in their initial volumes. However, its VRR after 1 year was not statistically different. Two studies revealed that VRR after RFA was superior in small nodules than in large ones (24, 27). Two studies showed that VRR after RFA was not correlated with the initial volume of TNs (18, 28). We used multivariate logistic and linear regression to exclude confounding factors, including gender, follow-up time, initial volume, number of TN, number of ablated nodules, history of RFA, history of thyroid surgery, and technical efficiency, to explore the effect of malignant nodules on volume and VRR after RFA. We saw that volume at years 1 and 2 after RFA in benign nodules was superior to malignant nodules, and its difference was statistically significant (p ≤ 0.001<0.05; p ≤ 0.001<0.05). Its VRR was also similar (p ≤ 0.001<0.05; p=0.037<0.05); moreover, its trend was consistent between the two. However, Yi L et al. found that the VRR after RFA for benign TNs was significantly lower in the hypercalcification group than in the microcalcification group at years 3, 4, and 5 after RFA (29). This discrepancy may be due to the fact that the difference between malignant and benign nodules is not just calcification.

The limitations of our study are as follows: 1. Our study is a single-center clinical study and its conclusions need to be validated in multicenter, large-sample studies. 2, our study only examined the effect of malignant nodules on the efficacy of RFA. The current increase in the indications for RFA and, therefore, the factors influencing its efficacy may increase, like nodules in residual glands after surgery, nodules after multiple ablations, and recurrent nodules. We will discuss these issues in subsequent studies.

## 5 Conclusion

Malignant thyroid nodules do not affect the efficacy of radiofrequency ablation. Although their early target regression is slower after RFA, there is no difference in long-term outcomes, and they are more likely to achieve complete regression.

## Data Availability

The raw data supporting the conclusions of this article will be made available by the authors, without undue reservation.

## References

[1] Trimboli P Castellana M Sconfienza LM Virili C Pescatori LC Cesareo R. Efficacy of thermal ablation in benign non-functioning solid thyroid nodule: A systematic review and meta-analysis. Endocrine. (2020) 67:35–43. doi: 10.1007/s12020-019-02019-3, PMID: 31327158

[2] Che Y Jin S Shi C Wang L Zhang X Li Y. Treatment of benign thyroid nodules: comparison of surgery with radiofrequency ablation. AJNR Am J Neuroradiol. (2015) 36:1321–5. doi: 10.3174/ajnr.A4276, PMID: 25814656 PMC7965284

[3] Kim YS Rhim H Tae K Park DW Kim ST. Radiofrequency ablation of benign cold thyroid nodules: initial clinical experience. Thyroid. (2006) 16:361–7. doi: 10.1089/thy.2006.16.361, PMID: 16646682

[4] Sim JS Baek JH. Long-term outcomes of thermal ablation for benign thyroid nodules: the issue of regrowth. Int J Endocrinol. (2021) 2021:9922509. doi: 10.1155/2021/9922509, PMID: 34335748 PMC8321738

[5] Yan L Zhang M Li X Li Y Luo Y. A nomogram to predict regrowth after ultrasound-guided radiofrequency ablation for benign thyroid nodules. Front Endocrinol (Lausanne). (2022) 12:774228. doi: 10.3389/fendo.2021.774228, PMID: 35250847 PMC8891142

[6] Bernardi S Cavallaro M Colombin G Giudici F Zuolo G Zdjelar A. Initial ablation ratio predicts volume reduction and retreatment after 5 years from radiofrequency ablation of benign thyroid nodules. Front Endocrinol (Lausanne). (2021) 11:582550. doi: 10.3389/fendo.2020.582550, PMID: 33597921 PMC7883676

[7] Bernardi S Giudici F Cesareo R Antonelli G Cavallaro M Deandrea M. Five-year results of radiofrequency and laser ablation of benign thyroid nodules: A multicenter study from the italian minimally invasive treatments of the thyroid group. Thyroid. (2020) 30:1759–70. doi: 10.1089/thy.2020.0202, PMID: 32578498

[8] Aljammal J Jasim S Alahdab F Rowley A Ferenczi A Schmeltz L. Predictors of initial response of thyroid nodules treated with RFA, a multi-endocrinology centers experience from the United States. J Endocr Soc. (2025) 9:bvaf077. doi: 10.1210/jendso/bvaf077, PMID: 40452798 PMC12123420

[9] Dobrinja C Bernardi S Fabris B Eramo R Makovac P Bazzocchi G. Surgical and pathological changes after radiofrequency ablation of thyroid nodules. Int J Endocrinol. (2015) 2015:576576. doi: 10.1155/2015/576576, PMID: 26265914 PMC4523654

[10] Kim MK Shin JH Hahn SY Kim H. Delayed cancer diagnosis in thyroid nodules initially treated as benign with radiofrequency ablation: ultrasound characteristics and predictors for cancer. Korean J Radiol. (2023) 24:903–11. doi: 10.3348/kjr.2023.0386, PMID: 37634644 PMC10462893

[11] Tessler FN Middleton WD Grant EG Hoang JK Berland LL Teefey SA. ACR thyroid imaging, reporting and data system (TI-RADS): white paper of the ACR TI-RADS committee. J Am Coll Radiol. (2017) 14:587–95. doi: 10.1016/j.jacr.2017.01.046, PMID: 28372962

[12] Mauri G Pacella CM Papini E Solbiati L Goldberg SN Ahmed M. Image-guided thyroid ablation: proposal for standardization of terminology and reporting criteria. Thyroid. (2019) 29:611–8. doi: 10.1089/thy.2018.0604, PMID: 30803397

[13] Ahn HS Kim SJ Park SH. Radiofrequency ablation of benign thyroid nodules: evaluation of the treatment efficacy using ultrasonography. Ultrasonography. (2016) 35:244–52. doi: 10.14366/usg.15083, PMID: 27101983 PMC4939722

[14] Kandil E Omar M Aboueisha M Attia AS Ali KM Abu Alhuda RF. Efficacy and safety of radiofrequency ablation of thyroid nodules: A multi-institutional prospective cohort study. Ann Surg. (2022) 276:589–96. doi: 10.1097/SLA.0000000000005594, PMID: 35837903

[15] Lim HK Cho SJ Baek JH Lee KD Son CW Son JM. US-guided radiofrequency ablation for low-risk papillary thyroid microcarcinoma: efficacy and safety in a large population. Korean J Radiol. (2019) 20:1653–61. doi: 10.3348/kjr.2019.0192, PMID: 31854153 PMC6923213

[16] Koike E Noguchi S Yamashita H Lee KD Son CW Son JM. Ultrasonographic characteristics of thyroid nodules: prediction of Malignancy. Arch Surg. (2001) 136:334–7. doi: 10.1001/archsurg.136.3.334, PMID: 11231857

[17] Park KW Shin JH Han BK Ko EY Chung JH. Inoperable symptomatic recurrent thyroid cancers: preliminary result of radiofrequency ablation. Ann Surg Oncol. (2011) 18:2564–8. doi: 10.1245/s10434-011-1619-1, PMID: 21347777

[18] Wu MH Chen KY Chen A Chen CN. Differences in the ultrasonographic appearance of thyroid nodules after radiofrequency ablation. Clin Endocrinol (Oxf). (2021) 95:489–97. doi: 10.1111/cen.14480, PMID: 33938024

[19] Baek JH Moon WJ Kim YS Lee JH Lee D. Radiofrequency ablation for the treatment of autonomously functioning thyroid nodules. World J surgery. (2009) 33:1971–7. doi: 10.1007/s00268-009-0130-3, PMID: 19575141

[20] Huh JY Baek JH Choi H Kim JK Lee JH. Symptomatic benign thyroid nodules: efficacy of additional radiofrequency ablation treatment session–prospective randomized study. Radiology. (2012) 263:909–16. doi: 10.1148/radiol.12111300, PMID: 22438360

[21] Yan L Zhang M Li X Li Y Luo Y. A nomogram to predict regrowth after ultrasound-guided radiofrequency ablation for benign thyroid nodules. Front Endocrinol (Lausanne). (2022) 12:774228. doi: 10.3389/fendo.2021.774228, PMID: 35250847 PMC8891142

[22] Sim JS Baek JH Lee J Cho W Jung SI. Radiofrequency ablation of benign thyroid nodules: depicting early sign of regrowth by calculating vital volume. Int J hyperthermia. (2017) 33:905–10. doi: 10.1080/02656736.2017.1309083, PMID: 28540795

[23] Zhang Y Zhang MB Luo YK Li J Zhang Y Tang J. Effect of chronic lymphocytic thyroiditis on the efficacy and safety of ultrasound-guided radiofrequency ablation for papillary thyroid microcarcinoma. Cancer Med. (2019) 8:5450–8. doi: 10.1002/cam4.2406, PMID: 31359613 PMC6746112

[24] Lim HK Lee JH Ha EJ Sung JY Kim JK Baek JH. Radiofrequency ablation of benign non-functioning thyroid nodules: 4-year follow-up results for 111 patients. Eur Radiol. (2013) 23:1044–9. doi: 10.1007/s00330-012-2671-3, PMID: 23096937

[25] Negro R Greco G Deandrea M Rucco M Trimboli P. Twelve-month volume reduction ratio predicts regrowth and time to regrowth in thyroid nodules submitted to laser ablation: A 5-year follow-up retrospective study. Korean J Radiol. (2020) 21:764–72. doi: 10.3348/kjr.2019.0798, PMID: 32410415 PMC7231608

[26] Lin WC Wang CK Wang WH Kuo CY Chiang PL Lin AN. Multicenter study of benign thyroid nodules with radiofrequency ablation: results of 762 cases over 4 years in Taiwan. J Pers Med. (2022) 12:63. doi: 10.3390/jpm12010063, PMID: 35055378 PMC8782025

[27] Deandrea M Garino F Alberto M Garberoglio R Rossetto R Bonelli N. Radiofrequency ablation for benign thyroid nodules according to different ultrasound features: an Italian multicentre prospective study. Eur J Endocrinol. (2019) 180:79–87. doi: 10.1530/EJE-18-0685, PMID: 30407921

[28] Deandrea M Trimboli P Garino F Mormile A Magliona G Ramunni MJ. Long-term efficacy of a single session of RFA for benign thyroid nodules: A longitudinal 5-year observational study. J Clin Endocrinol Metab. (2019) 104:3751–6. doi: 10.1210/jc.2018-02808, PMID: 30860579

[29] Li Y He H Li W Zhao J Ge N Zhang Y. Efficacy and safety of radiofrequency ablation for calcified benign thyroid nodules: results of over 5 years’ follow-up. BMC Med Imaging. (2022) 22:75. doi: 10.1186/s12880-022-00795-5, PMID: 35459125 PMC9027040

